# Experimental approach to study the effect of mutations on the protein folding pathway

**DOI:** 10.1371/journal.pone.0210361

**Published:** 2019-01-14

**Authors:** Elena V. Nemtseva, Marina A. Gerasimova, Tatiana N. Melnik, Bogdan S. Melnik

**Affiliations:** 1 Siberian Federal University, Krasnoyarsk, Russia; 2 Institute of Biophysics, Siberian Branch of Russian Academy of Sciences, Krasnoyarsk, Russia; 3 Institute of Protein Research, Russian Academy of Sciences, Pushchino, Moscow Region, Russia; University of Milano-Bicocca, ITALY

## Abstract

Is it possible to compare the physicochemical properties of a wild-type protein and its mutant form under the same conditions? Provided the mutation has destabilized the protein, it may be more correct to compare the mutant protein under native conditions to the wild-type protein destabilized with a small amount of the denaturant. In general, is it appropriate to compare the properties of proteins destabilized by different treatments: mutations, pH, temperature, and denaturants like urea? These issues have compelled us to search for methods and ways of presentation of experimental results that would allow a comparison of mutant forms of proteins under different conditions and lead to conclusions on the effect of mutations on the protein folding/unfolding pathway. We have studied equilibrium unfolding of wild-type bovine carbonic anhydrase II (BCA II) and its six mutant forms using different urea concentrations. BCA II has been already studied in detail and is a good model object for validating new techniques. In this case, time-resolved fluorescence spectroscopy was chosen as the basic research method. The main features of this experimental method allowed us to compare different stages of unfolding of studied proteins and prove experimentally that a single substitution of the amino acid in three mutant forms of BCA II affected the native state of the protein but did not change its unfolding pathway. On the contrary, the inserted disulfide bridge in three other mutant forms of BCA II affected the protein unfolding pathway. An important result of this research is that we have validated the new approach allowing investigation of the effect of mutations on the folding of globular proteins, because in this way it is possible to compare proteins in the same structural states rather than under identical conditions.

## Introduction

To appreciate the relationship between the amino acid sequence of the protein and its 3D structure it is of importance to study the succession of formation/destruction of different structure elements for the protein folding/unfolding (the protein unfolding pathway). There are a great number of theoretical works where the protein folding pathway has been appraised and analyzed, for example, [1]. It was shown that substitutions of amino acid residues, truncation/elongation of the loops and structure elements can affect not only the protein stability but also change completely its folding pathway, i.e., the sequence of formation of different protein regions, for example, [2–6]. Unfortunately, only few experimental methods and techniques are at hand that allow studying the protein folding pathway. It is not surprising that to study the protein folding it is critical to obtain data on separate parts of the polypeptide chain rather than typical characteristics of the whole protein. Imagine that we investigate two types of mutant forms of the same protein. In the first case, a single substitution of the amino acid residue has been made, and in the second case a cysteine bridge connecting two structure elements remote along the amino acid sequence has been introduced. Both mutations can stabilize or destabilize the protein uniformly, but would they change the protein folding pathway? Naturally it is clear that a single substitution of the amino acid residue should not affect the succession of assembly or destruction of structure elements of the protein (its folding pathway) and the insertion of the cysteine bridge can change the pathway. However are there experimental methods that could substantiate or negate our supposition? At present only two methods that can be used to solve this problem are available.

The first is the so-called “Φ-value analysis”. It is an approach allowing experimental identification of the amino acids “responsible” for the formation of the transient protein state. The principle of the method is to study the folding and unfolding kinetics of numerous (up to several dozen) mutant forms of protein with single substitutions of amino acids. As a result, such studies can clarify what amino acid residues are involved in the formation of the protein transient state [5,7–10].

The other approach is the analysis of the extent of deuteration of different regions of the polypeptide chain of protein upon its folding/unfolding using the NMR and mass spectrometry methods. As a rule, three groups of amino acids whose deuteration proceeds for about several microseconds, milliseconds and seconds are identified. Respectively, it is possible to identify protein chain regions that are formed at different stages of the protein folding (see, for example, [11–16]).

In addition to the above-described methods, there is a relatively informative and simple from the experimental point of view approach that enables studies of key stages of protein folding/unfolding with the use of the fluorescence and circular dichroism techniques. The use of this approach requires plotting interdependences of different spectral parameters along with usual plots of fluorescence intensity (or ellipticity) dependences on the denaturant concentration when studying equilibrium protein unfolding. This means that it is necessary to eliminate the “denaturant concentration” parameter and plot, for example, the dependence of fluorescence intensity at 320 nm (I_320_) versus the fluorescence intensity at 360 nm (I_360_) [17–19].

Graphs of interdependence of different spectral parameters (fluorescence, CD, FTIR, absorption, etc.) can be used to reveal the presence of intermediate states upon equilibrium protein unfolding. The analysis of such dependences is rather simple. If at an increase in the denaturant concentration the whole protein structure changes, different parameters characterizing the protein state should change “synchronously”. But if different structure elements of the protein unfold non-simultaneously, for example, because of the domain structure or due to the formation of intermediate states, the spectral parameters would also change “at a different rate”. Thus for a protein which folds/unfolds in a single stage such dependences are always straight lines. For multidomain proteins or proteins folding in multiple stages, such dependences are lines with sharp bends or turns. The theoretical background and examples of analysis of such dependences can be found elsewhere [17–19].

Plots of the dependence of a spectral parameter on another spectral parameter can be used to identify the number of intermediate states but cannot be used for studying the protein folding pathway. The point is that the steady-state fluorescence measurements give the spectral parameters in relative units. So, the steady-state parameters depend not only on the protein structure but also on its concentration. Moreover, the fluorescence spectra can also be distorted by wavelength-dependent efficiency of the emission monochromator and detector, absorption of the sample, background scattering and fluorescence, *etc*. [20]. Therefore it is difficult to compare the plots of spectral parameters, for example, I_320_ versus I_360_, for different proteins if fluorescence was measured using different types of spectrofluorometers unless a correction of all distortions has been performed properly. One should also note that the fluorescence spectrum of multi-tryptophan protein is a sum of several emission bands [21]. In this case the choice of the most informative wavelength(s) to follow the folding/unfolding process is not a trivial task.

In this study, we used the time-resolved fluorescence spectroscopy technique to analyze the BCA II folding/unfolding pathway. It allows examining the excited-state lifetimes and their contributions into total fluorescence of the protein after ultrashort-pulse laser excitation [20–23]. Protein fluorescence under excitation at >290 nm is mainly contributed by tryptophan residues whose fluorescence is highly sensitive to the local environment and, consequently, to the change in protein conformation [21]. In contrast to fluorescence intensity measured by the steady-state technique the fluorescence lifetimes are independent of the protein concentration in the sample, light scattering and many potential instrumental artifacts that can distort the data [20,21,24]. So an advantage of the time-resolved fluorescence spectroscopy technique in application to proteins is that the excited state lifetimes of a tryptophan residue can be considered as an absolute measure. An additional important point is that we used the global analysis of lifetimes determined by fitting the set of fluorescence decays over the whole emission range of the protein (300–430 nm) [25–28]. It means that the obtained time-resolved fluorescence parameters of the proteins were averaged over the whole emission spectra and thus independent of the emission wavelength as well.

In our previous study we demonstrated that application of the fluorescence lifetimes revealed the intermediate states of the model protein during equilibrium denaturation that were “invisible” from the steady-state spectral data [27]. In this work we have used the peculiarities of the time-resolved fluorescent spectroscopy method to study the folding/unfolding pathway of different mutant forms of globular proteins. In particular, the effect of single amino acid substitution and insertion of disulfide bridge to the bovine carbonic anhydrase II folding/unfolding has been analyzed.

## Result and discussion

### Choice of research method

**Fig 1:**
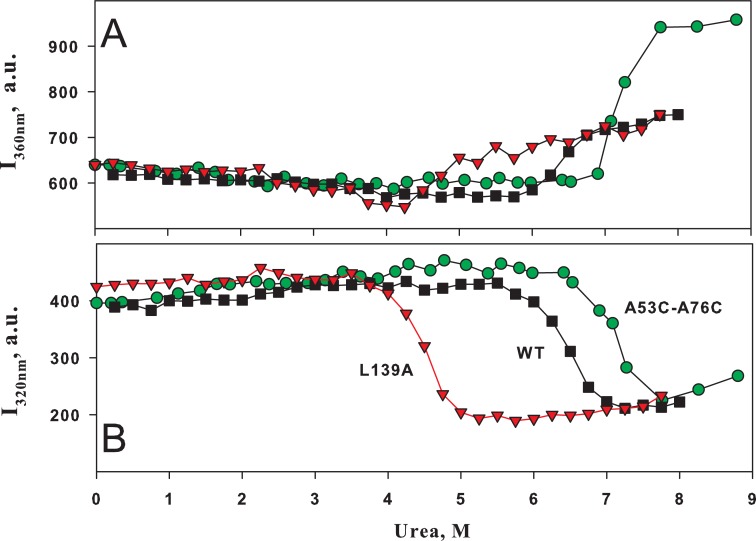
Effect of urea on fluorescence intensity of BCA II. Dependence of fluorescence intensity **I** at 360 nm (A) and 320 nm (B) on urea concentration for wild-type BCA II (■ WT) and its two mutant forms (▼ L139A and ● A53C-A76C). Excitation at 280 nm. The vertical line at 7 M is a reference to compare the curves (see the text).

The aim of our study was to choose an experimental approach that would allow a comparison of the folding/unfolding pathway of different mutant forms of the protein and to analyze the effect of amino acid substitutions on the succession of formation of protein structure elements. Unfortunately this cannot be achieved using typical spectral methods (for example, steady-state fluorescence and circular dichroism) combined with usual plots of dependence of spectral parameters on the denaturant concentration. Thus, in our previous papers we used dependences of ellipticity and fluorescence intensity at fixed wavelengths on urea concentration to study wild-type BCA II and its mutant forms [29–31]. Those plots allowed us to analyze the presence and stability of intermediate states in each protein (for example, see Fig 1), but they could not explain in what way the mutations affected the folding pathway of this protein. The folding pathway is a chain of changes in the protein structure not always connected with its stability. Provided we have changed the stability of a protein state, but after plotting a typical graph (the dependence of a spectral parameter on the denaturant concentration) we will see shifted transition curves, though the folding pathway will not change.

Accordingly, to study the protein folding it is required to have graphs in which there are no parameters connected with the experiment conditions but only parameters related to the protein structure and properties. Therefore, the idea that *it is necessary to eliminate the “denaturant concentration” parameter* has emerged. It is not novel. The team from Prof. Turoverov’s Laboratory performed many studies of different proteins and showed that diagrams plotted using two spectral parameters, excluding the denaturant concentration parameter, are far more informative when the number of intermediate states generated upon protein unfolding is determined [17–19].

To begin with, we used this idea for wild-type BCA II and its two distinct mutant forms, L139A and A53C-A76C. L139A is a mutant with a single amino acid substitution which essentially destabilized the protein [25,28]. The double substitution, A53C and A76C, was performed to insert an additional disulfide bridge to BCA II. This mutation stabilized the protein [27]. As demonstrated by the kinetic experiments, each mutation affected the early and later stages of carbonic anhydrase II folding/unfolding in a different way. The analysis of the chevron plots and rate constants obtained upon nonequilibrium melting of BCA II led to conclusion that the L139A substitution did not change the carbonic anhydrase II folding pathway whereas the disulfide bridge changed it [27].

Fig 1 shows dependences of fluorescence intensity of three proteins on urea concentration used when studying equilibrium unfolding of proteins. The comparison of curves in Fig 1A and 1B indicates the formation of intermediate states upon protein unfolding because equilibrium transition midpoints are not compatible. This is clearly seen in the case of double mutant form A53C-A76C. For this protein, in Fig 1A the equilibrium transition just starts at 7 M urea, while in Fig 1B this concentration is about the midpoint of the transition. Though the plots in Fig 1A and 1B contain definite information about intermediate protein states, it is difficult to analyze how many intermediate states exist upon BCA II unfolding.

**Fig 2:**
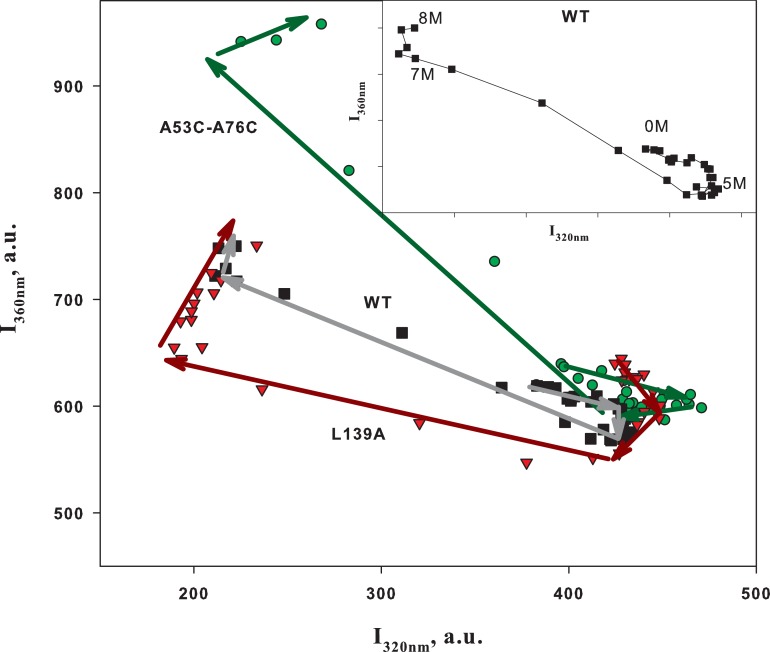
Phase diagram analysis of urea-induced unfolding of BCA II. Interdependence of fluorescence intensities at 320 and 360 nm for wild-type BCA II (■ WT) and its two mutant forms (▼ L139A and ● A53C-A76C) measured at different urea concentrations. Excitation at 280 nm. Arrows show the increase in urea concentration and accordingly the sequence of protein unfolding. Insertion: The dependence for the wild-type protein (WT) is shown with indication of urea concentrations in “key” points of the plot.

To make the analysis of such data more convenient, we plotted interdependences of different spectral parameters as it was proposed earlier [18]. Fig 2 demonstrates the diagram of fluorescence intensity at 360 nm (I_360_) *versus* fluorescence intensity at 320 nm (I_320_) for wild-type BCA II and its two mutant forms. Each dependence is a sharply bent or broken curve. The number of sharp bends on such curves should be associated with the number of intermediate states upon protein folding [17–19]. Theoretical background and analysis of such plots are described in detail elsewhere [17]. The dependences in Fig 1 show that there are several protein states but it is difficult to “see” them, while the curves in Fig 2 visualize this information. In particular, the analysis of the curve shapes in Fig 2 suggests that upon unfolding of wild-type BCA II and its two mutant forms at least two intermediate states are generated. We can see transitions between these states as linear regions in Fig 2. The insertion in Fig 2 shows the curve for wild-type BCA II with marks indicating the urea concentrations where the plot is “broken”.

It should be reminded that the purpose of our research is to obtain information about the influence of mutations on the protein folding pathway. It was found that from this point of view, the plots in Fig 2 cannot be used for analysis of the protein folding pathway. Fig 2 led us to an unambiguous conclusion that most likely the unfolding of the wild-type protein and its two mutant forms gives rise to the same number of intermediate states. It may be assumed that mutation L139A has not changed the unfolded protein state, while the disulfide bridge in A53C-A76C has changed. However these are only assumptions because the three curves differ from each other. It is not clear whether the differences in the curves of Fig 2 can be connected with the changes in the structure of different protein states upon its unfolding, i.e., with the change in the protein folding pathway.

It may seem that we have chosen an “incorrect” method or that the measurements should have been made in more detail and more accurately. Unfortunately this is not so! A similar result will be obtained with the use of any method notwithstanding the accuracy of the measurements. The point is that the differences in the curves of Fig 2 are caused not only by the different structures of intermediate states but to a greater extent by different populations of the intermediate states in different proteins.

Thus, *if it is required to plot graphs or diagrams connected with the protein folding pathway*, *it is necessary to use parameters independent of the population of different protein states and dependent only on the conformation of the protein polypeptide chain*. It is extremely difficult to retrieve such parameters because any spectra (of fluorescence, absorption, circular dichroism etc.) undoubtedly depend both on the protein concentration in the preparation and the populations in different protein states. And if it is possible to control the general protein concentration, it is very difficult to measure the population in a protein state and in most cases is merely unattainable.

From the experimental point of view, there is only one simple and accessible parameter independent of either concentration or population of intermediate states–it is the lifetime of the excited state of chromophores. In the case of proteins, it is the lifetime of the excited state of tryptophan residues (τ). On the one hand, this parameter is highly sensitive to the surrounding of the tryptophan residue, i.e. on the conformation of the polypeptide chain. On the other hand, in contrast to fluorescence intensity, it is independent of either the protein concentration or the populations in different protein states. Though, of course, it has disadvantages. The τ parameter, the lifetime of the excited state of tryptophan residues, can be used only when studying proteins, the structure of which contains several tryptophan residues located in different protein structure elements. This means that it is necessary to be certain that the τ parameter would really allow obtaining information about the changes in the protein structure. The second disadvantage that limits fluorescence lifetimes application in this field is the difficulty in connection of obtained discrete lifetimes with the individual tryptophan residues in protein structure. Tryptophan fluorescence decays display, as a rule, three lifetimes independently of whether this amino acid is free in solution or is included into the structure of single- or multi-tryptophan protein [22,23,27,28]. So the fluorescence lifetime components of multi-tryptophan proteins can be assumed as some integral parameters reflecting the structure peculiarities of the protein under the given equilibrium conditions.

### A fluorescence lifetime of tryptophan residues as informative parameter to study the protein folding pathway

As shown in our previous paper [27], the data on BCA II unfolding obtained by the means of time-resolved fluorescent method under equilibrium conditions agree well with the parameters of chevron plots created by the results of kinetic experiments [30,31]. Thus it has become clear that lifetimes of the excited state of tryptophan residues and its contribution to the steady-state fluorescence intensity at different denaturant concentrations contain information about intermediate states of BCA II. Indeed, the amino acid sequence of BCA II has seven amino acid tryptophan residues. They are located in different structure elements of the protein: some are closer to the surface of the protein and some are deeply buried in its hydrophobic core [27].

In our experiments, the fluorescence decay curves were measured throughout the emission spectra for the BCA II and its mutant forms after incubation with different urea concentrations. Two lifetime components, τ_1_ (4.7–5.9 ns) and τ_2_ (1.1–2.0 ns), were determined for each decay set by the means of the global analysis. The details on experimental procedure and data processing can be found in the Materials and Methods section. The obtained ranges of values of τ_1_ and τ_2_ are quite typical of protein fluorescence [21,23,27,28], but in contrast most studies they are characteristic of overall fluorescence spectra and not the emission at one wavelength of the sample.

**Fig 3:**
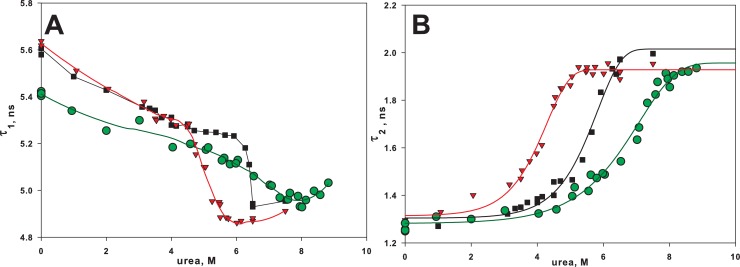
Effect of urea on fluorescence lifetimes of BCA II. Dependence of fluorescence lifetimes τ_1_ (A) and τ_2_ (B) on urea concentration of wild-type BCA II (■ WT) and its two mutant forms (▼ L139A and ● A53C-A76C). The relative deviations for τ_1_ and τ_2_ were ≤3% and ≤7% correspondingly. The solid lines are to guide the eyes.

**Fig 4:**
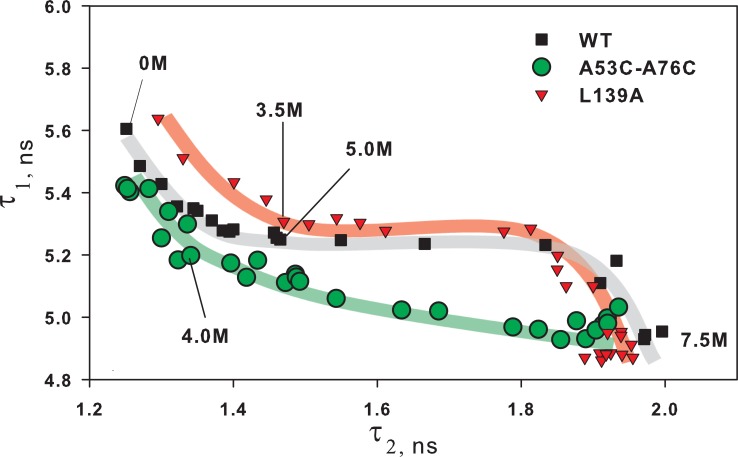
Phase diagram of two fluorescence lifetime components. Interdependence of two fluorescence lifetime components τ_1_ and τ_2_ of wild-type BCA II (■ WT) and its two mutant forms (▼ L139A and ● A53C-A76C). Each point was obtained by fitting fluorescence decay curves of protein solution at definite urea concentration. Thick color lines show the “unfolding pathway” for each protein with increase in urea concentration from 0 to 7.5 M. The relative deviations for τ_1_ and τ_2_ were ≤3% and ≤7% correspondingly.

Dependences of the parameters τ_1_ and τ_2_ on urea concentration are given in Fig 3. Fig 4 shows the diagram of τ_1_ versus τ_2_.

Fig 3 is a more typical graph. It allows us to make conclusions on stabilization or destabilization of proteins but not on the effect of mutations on the unfolding pathway, because such coordinates as the spectral parameter versus the denaturant concentration enable comparing proteins under the same conditions rather than proteins in the same structural conformations.

As described above, parameters τ are independent of either the protein concentration or population of its intermediate states but depend on the protein structure. Therefore it is possible to interpret Fig 4 in the following way. Each point in the plot plane means a certain protein state with a combination of τ_1_ and τ_2_ characteristic of only this state. It is possible to explain this plot by comparing it with two-dimensional electrophoresis where proteins are separated according to their charge in one direction and according to their size in the other. In the case of electrophoresis, it is sufficient to have two “axes” to distinguish most proteins. In our case, it is enough to have two parameters τ to distinguish proteins with different conformations generated upon their unfolding.

The color lines in Fig 4 drawn by the experimental points can be interpreted as a sequence of different structural states of each protein generated upon their unfolding, from 0 M to 7.5 M urea, i.e. the unfolding pathway.

As seen from Fig 4, the wild-type protein and the protein with the substitution L139A differ mainly at low urea concentrations. And when both proteins are destabilized their “pathways” coincide. This is quite logic if we assume that a single substitution of the hydrophobic amino acid (L139A) has destabilized the protein but has not changed essentially its structure. Destabilization has affected the rigidly packed native state but has not changed the subsequent mobile states of the protein.

Analogous considerations can be used in the analysis of the influence of the disulfide bond on the unfolding pathway of BCA II. A specially inserted cysteine bridge (A53C-A76C) to the protein surface has no strong effect on the structure of the native state of BCA II, but it should affect the unfolding because it “strengthens” structure elements that unfold in the wild-type protein. Just in this way we can interpret the curve in Fig 4 for protein A53C-A76C. At low urea concentration the curves for A53C-A76C and WT proteins are similar and at high concentration they “diverge”.

### Some speculations

As opposed to typical graphs (Figs 1–3), Fig 4 permits us to get answers to some of the challenges posed in the Abstract. For example, is it possible to compare the protein destabilized by mutation with the protein destabilized by a denaturant? The answer is yes and no! It was found that all depends on the type of mutation and conditions under which we would like to compare the proteins. For example, it is impossible to compare wild-type BCA II and the protein with a single amino acid substitution L139A under native conditions (at low concentrations of the denaturant), but is possible at some urea concentrations. The reason is that at high concentrations of the denaturant the both proteins “pass” the same unfolding pathway, i.e. they have the same structural conformations. For example, protein L139A in 3.5 M urea has the same conformation as the wild-type protein in 5 M urea (see Fig 4).

In contrast to the above, A53C-A76C mutant form with the inserted disulfide bridge on its surface can be comparable to the wild-type protein only at a low denaturant concentration however upon further unfolding the pathways of these proteins diverge.

### Check of a research method using several mutant forms of BCA II

The approach proposed in this work is novel, and it definitely requires numerous tests and comparisons with other experimental methods. To ensure the sensitivity of our research method to alterations of folding/unfolding pathway, we studied three mutant variants of carbonic anhydrase with single amino acid substitutions and three mutants with inserted disulfide bridges. It was intuitively clear that single substitutions could not have much influence on folding/unfolding pathway, while disulfide bridges could change it dramatically.

**Fig 5:**
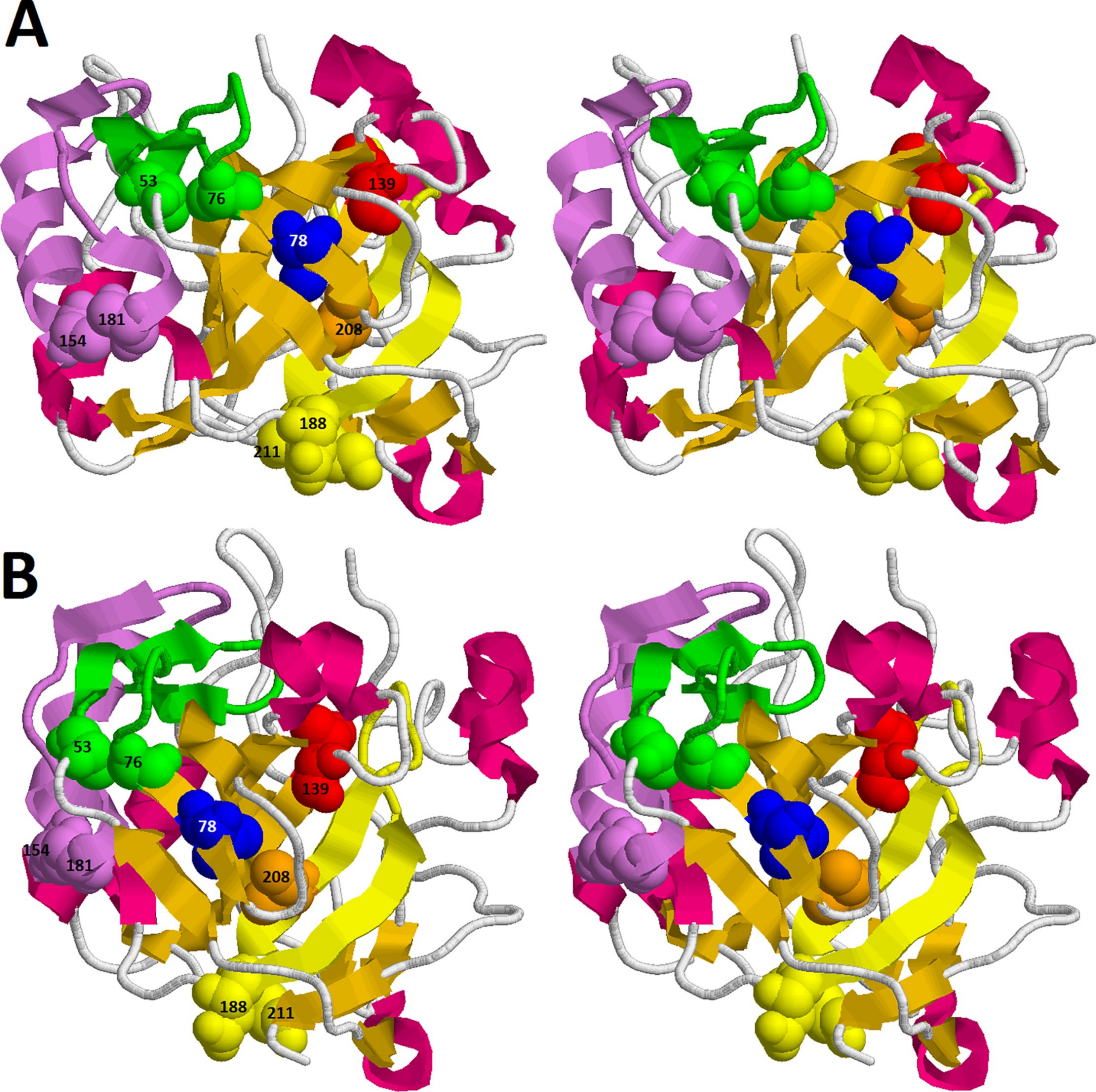
Three-dimensional structure of BCA II. (A) and (B) stereo view differ in the small turn of protein allowing to analyze the position of substituted amino acids (balls denoted). Three structure elements, αβ-hairpin and two β-hairpins, were cross-linked in A53C-A76C, A154C-S181C and D188C-K211C mutant forms by disulfide bridges colored green, purple and yellow correspondingly. Numbers of the substituted amino acids are indicated.

When designing disulfide bridges, we chose amino acids in order to fix small structural elements, which, as we supposed, could be formed at different stages of carbonic anhydrase folding/unfolding. Fig 5 shows structure of carbonic anhydrase with highlighted mutated positions. An αβ-hairpin, which is fixed by S-S bond between residues 154 and 181 (shown in violet on Fig 5), is positioned on the protein surface, and it holds the following β-hairpin (green on Fig 5), which is constrained by A53C-A76C mutation. β-hairpin with D188C-K211C mutation is situated in the center of the protein and it is a part of central β-sheet of carbonic anhydrase. Thus, we hoped that A154C-S181C, A53C-A76C and D188C-K211C mutations would affect different stages of carbonic anhydrase unfolding, which could be seen on parametric plots. For single amino acid substitutions, hydrophobic amino acids situated in different structural elements of the hydrophobic core were chosen.

**Fig 6:**
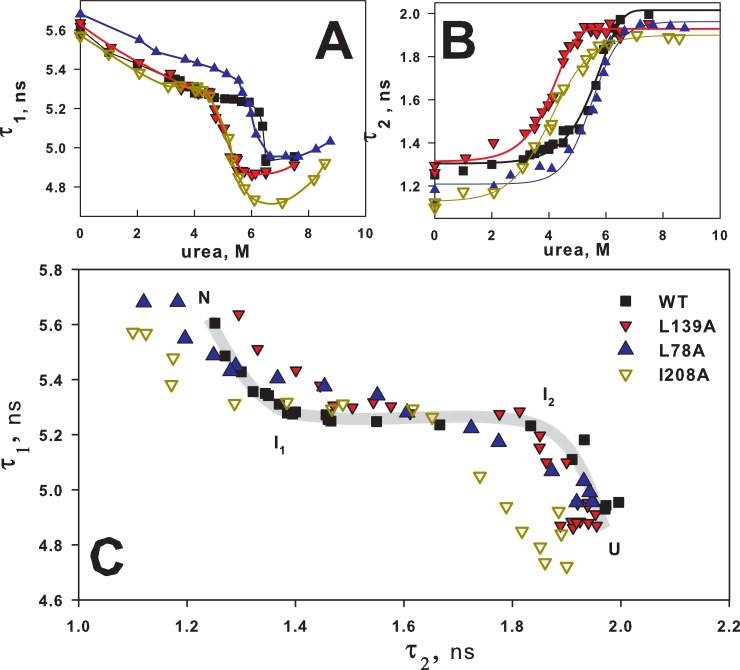
Effect of single substitution of amino acids on BCA II unfolding. Dependence of fluorescence lifetimes τ_1_ (A) and τ_2_ (B) on urea concentration of wild-type BCA II (■ WT) and its three mutant forms L78A (▲), L139A (▼), I208A (▽). (C)–interdependence of two fluorescence lifetime components τ_1_ and τ_2_ of wild-type BCA II and its three single substituted mutant forms. The relative deviations for τ_1_ and τ_2_ were ≤3% and ≤7% correspondingly.

**Fig 7:**
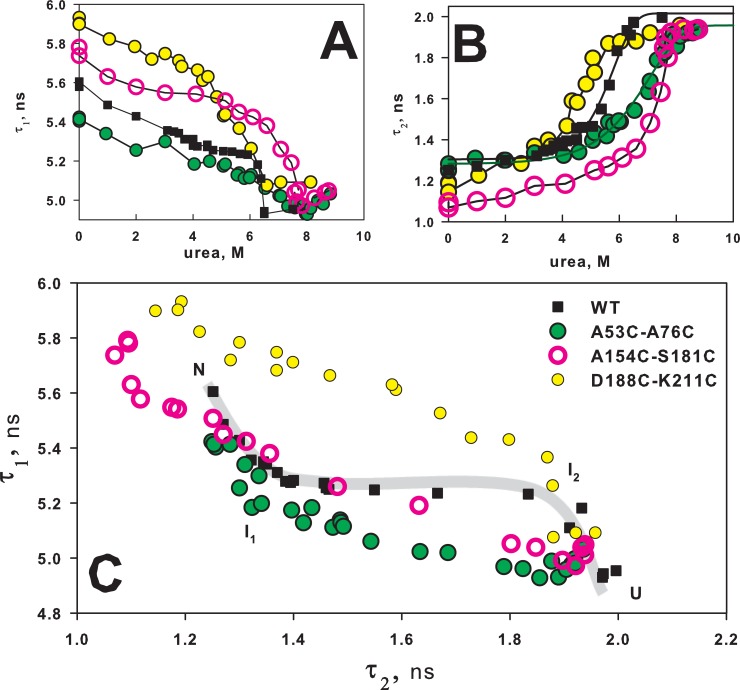
Effect of inserted disulfide bridge on BCA II unfolding. Dependence of fluorescence lifetimes τ_1_ (A) and τ_2_ (B) on urea concentration of wild-type BCA II (■ WT) and its three mutant forms with disulfide bridge A53C-A76C (●), A154C-S181C (○) and D188C-K211C (●). (C)–interdependence of two fluorescence lifetime components τ_1_ and τ_2_ of wild-type BCA II and its three mutant forms with inserted disulfide bridge. The relative deviations for τ_1_ and τ_2_ were ≤3% and ≤7% correspondingly.

Fig 6 shows fluorescence lifetimes data for mutant carbonic anhydrase forms with single amino acids substitutions, Fig 7 shows these data for proteins with cysteine bridges. Figs 6A, 6B, 7A and 7B show «conventional» dependencies of lifetime components on urea concentrations. These plots serve well to illustrate and discuss influence of mutations on stability of native or intermediate protein states, but it is difficult to explain these plots from the points of view of the impact of mutations on protein folding/unfolding pathway. Figs 6C and 7C, on the contrary, could be interpreted quite easily from the point of view of folding/unfolding pathway.

Fig 6C shows the diagram of τ_1_ versus τ_2_ for mutant carbonic anhydrase forms with single amino acid substitutions. All the curves have similar shape, which could be interpreted as protein transition between N, I_1_, I_2_, U states. Let us recall that transition between two states looks like a linear region on such a plot and every bend is additional intermediate state of protein [17–19]. Thus, we can suppose that L78A and L139A substitutions did alter just native protein state, and after its disruption the unfolding process passes through the same conformational states. I208A substitution could possibly affect not only the native state, but also the second intermediate state (I_2_), changing the part of protein unfolding pathway from I_2_ to the unfolded state (U).

Contrary to single substitutions, all the disulfide bridges changed carbonic anhydrase folding/unfolding pathway. τ_1_ versus τ_2_ diagrams on Fig 7C for all the mutant forms differ from wild-type protein in shape and number of bends corresponding to intermediate protein states. Our proposal on influence of the designed S-S-bridges on different carbonic anhydrase folding/unfolding stages was also proven. At least, it can be seen for proteins A53C-A76C and D188C-K211C. For A53C-A76C mutant: the initial stage of unfolding (N ↔ I_1_) looks similar to wild-type protein, but then the folding/unfolding processes of mutant protein and wild-type protein follow different ways. For D188C-K211C protein it is just the opposite: the final unfolding stage (I_2_↔ U) looks like the same stage in wild-type protein, and all the other parts of protein folding/unfolding process pass through different conformational states. τ_1_ versus τ_2_ diagram for A154C-S181C mutant is more difficult to interpret. The shape of this dependence does not look similar to the curve of wild-type protein. The disulfide bridge A154C-S181C could probably constrain a structural element which is unfolded first and could thus change the whole sequence of conformational states of carbonic anhydrase during unfolding. Of course, understanding the peculiarities of such conformational transformations of the protein requires additional studies, but studying carbonic anhydrase is not the goal of the current work. We just tried to demonstrate the informativeness of fluorescence lifetime components and plots like 6C and 7C.

## Conclusions

To study the protein folding pathway it is required to consider experimental parameters that are not related to the experiment conditions but only to the protein structure and properties. In our study we demonstrate that the diagrams reflected the change the fluorescence lifetime components of the protein during denaturation can be used for this purposes.

The chosen research method and the way of data interpretation allow us to compare the proteins in the same structural states rather than under the same conditions. In our opinion, the obtained results and the proposed approach make it possible for the first time to study the effect of mutations on the protein folding/unfolding pathway using experimental methods.

There is no doubt that the approach presented in this work requires testing on various proteins with different mutations. But, from our point of view, the number of mutant forms studied in the current work is enough to demonstrate the convenience and informativeness of interdependence of two fluorescence lifetime components (like 6C and 7C) during studying folding/unfolding pathway of globular proteins.

## Material and methods

### Protein expression and purification

The bovine carbonic anhydrase II coding sequence was reverse-transcribed from total RNA of bone marrow cells. cDNA was synthesized using gene specific oligonucleotide primer 3`-CA2 (5`-tttgtcgacGGCCAGTTCACCAAGTGGACTTGTG-3`(SalI restriction site is underlined) and M-MuLV reverse transcriptase (Fermentas, Lithuania). Products of the first strand synthesis were amplified using the polymerase chain reaction and gene specific oligonucleotide primer 5`-CA2 (5`-tacttttcatATGTCCCATCACTGGGGATAC-3`) (NdeI restriction site is underlined). The amplified carbonic anhydrase II gene was double digested with SalI and NdeI and inserted into the pET-11c_joe vector between NdeI and SalI restriction sites. The resulting plasmid was designated as pBCAB. Plasmids with the mutant BCA II genes were constructed by a standard PCR technique, using appropriate primers and a pET-28a vector as a template and a QuikChange kit (Stratagene, USA). The DNA sequences of all constructs were confirmed by the DNA sequence analysis. Carbonic anhydrase II and its mutant forms were expressed in *E*. *coli* cells and isolated as described elsewhere [32, 33].

### Protein chemistry

Disulfide bond formation in A53C-A76C, A154C-S181C and D188C-K211C mutant forms was performed as described in [34]. Then the quantity of free SH groups was defined by Ellman’s reagent [35].

Equilibrium denaturation was performed by incubating the proteins for not less than 12 h at 20°C in 20 mM Tris-HCl buffer, pH 8, containing increasing concentrations of urea (0–7.5 M). The final concentration of urea was defined from the refractive index measured by IRF-454-B2 M (KOMZ, Kazan, Russia).

The protein concentration was determined by UV absorption at 280 nm with the extinction coefficient *A* ^0.1%^_280_ = 1.87 [36,37].

The UV absorption spectra were measured with a Cary 5000i spectrophotometer (Agilent Technologies, Australia).

### Steady-state and time-resolved fluorescence measurements

Steady-state fluorescence of the proteins were measured using Cary 100 (Varian, Australia) and Fluorolog 3–22 (Horiba Jobin Yvon, USA) spectrofluorometers with standard 1 cm path-length quartz cuvettes. The excitation wavelengths were 280 and 296 nm. The emission spectra were recorded in the range 300–500 nm at the protein concentration of 0.1 mg/ml. All spectra were corrected for the background intensities and for the inner filter effect.

Time-resolved fluorescence measurements were performed using a Fluorolog 3–22 spectrofluorometer (Horiba Jobin Yvon, USA) equipped with a DeltaHub timing module for time-correlated single photon counting. A NanoLED pulsed diode (296 nm, the pulse duration of ~ 1.2 ns) was applied as an excitation source. Time-resolved fluorescence decays were collected in the range of 305–419 nm with an increment of 3 nm and time resolution of 27 ps/channel.

In order to recover the fluorescence decay parameters (amplitudes and lifetimes), the global analysis approach [25,26] was performed using the deconvolution DAS6 software (Horiba). The set of 39 decays collected for each sample was fitted with one, two, three and four lifetimes. The fit quality was evaluated by its global χ^2^ value and weighted residuals plot. It was found that for all samples the model with three exponential components gives the best fitting of the experimental data (see [27]). As a result, the time-resolved fluorescence decay at the wavelength λ was described as a sum of three exponents:

$$I_{\lambda }(t)=\sum_{i=1}^{3}\alpha_{i}^{\lambda }\;\exp(-t/\tau_{i}),$$

where *τ_i_* is the lifetime and $\alpha_{i}^{\lambda }$ is the amplitude of the *i* component.

The wavelength-dependent fractional coefficient $f_{i}^{\lambda }$ of each lifetime component was determined as

$$f_{i}^{\lambda }=\frac{\alpha_{i}^{\lambda }\;\tau_{i}}{\sum_{i}\alpha_{i}^{\lambda }\;\tau_{i}}.$$

Then the normalized contribution of the *i* component to the steady-state fluorescence intensity *A_i_* was calculated as

$$A_{i}=\frac{\sum_{\lambda }I_{\mathrm{ss}}^{\lambda }\;f_{i}^{\lambda }}{\sum_{\lambda }I_{\mathrm{ss}}^{\lambda }\;},$$

where $I_{\mathrm{ss}}^{\lambda }$ is the steady-state fluorescence intensity at wavelength λ. The contribution of the short lifetime (τ_3_<0.25 ns) was found to be small (A_3_<0.1) which was the reason for excluding it from consideration.

The results were processed and analyzed using the Microsoft Excel and Microcal Origin Pro 8.1 software.

### Reversibility of folding/unfolding of BCA II and its mutant forms in equilibrium experiments

For check of reversibility of WT and mutant forms of BCA II the technique described earlier in [38] has been used. The proteins were incubated for 48 hours in buffer solution with 8M urea and then dialyzed against Tris HCl buffer, pH 8.0 to achieve native conditions. After that, circular dichroism spectra were taken and protein melting was studied. As spectra and melting curves were the same as for non-denatured proteins, a conclusion was made that unfolding of wild-type carbonic anhydrase and its mutant forms is reversible.

The authors have no conflict of interest to declare.

## Abbreviations

BCA II: Bovine carbonic anhydrase II
CD: circular dichroism
FTIR: Fourier-transform infrared spectroscopy
NMR: Nuclear magnetic resonance
